# Malignant melanoma metastasis in the gallbladder. A case report of an unusual metastatic site

**DOI:** 10.1016/j.ijscr.2020.09.116

**Published:** 2020-09-19

**Authors:** Gabriel Fridolin Hess, Katharina Glatz, Sacha I. Rothschild, Otto Kollmar, Savas Deniz Soysal, Daniel T. Boll, Raoul André Droeser, Robert Mechera

**Affiliations:** a Clarunis University Center for Gastrointestinal and Liver Disease, St. Clara Hospital and University Hospital Basel, Spitalstrasse 21, CH-4056, Basel, Switzerland; b Institute of Pathology, University Hospital Basel, Schönbeinstrasse 40, CH-4056, Basel, Switzerland; c Department of Internal Medicine, Medical Oncology, University Hospital Basel, Switzerland Petersgraben 4, CH-4051, Basel, Switzerland; d Department of Radiology and Nuclear Medicine, University Hospital Basel, Switzerland Petersgraben 4, CH-4051, Basel, Switzerland

**Keywords:** MDT, multidisciplinary team meeting, OS, overall survival, MEK, mitogen-activated protein kinase, PET, positron emission tomography, CT, computed tomography, Malignant melanoma, Gallbladder metastasis, Cholecystectomy, Immunotherapy, Case report

## Abstract

**Introduction:**

Malignant melanoma is a neoplasia with the ability to metastasize to all organs. Most frequently, metastases derives from a skin primary. A solitary metastasis in the gallbladder is rarely mentioned in current literature.

**Presentation of case:**

We present the case of a 62-year-old female patient with the unusual metastatic spread of malignant melanoma into the gallbladder. The lesion was detected during routine follow up appointment six years after the initial surgical and radio-chemotherapeutic treatment of a malignant melanoma on the back. Following multidisciplinary team meeting, it was decided to perform a laparoscopic cholecystectomy to remove the gallbladder metastasis.

**Discussion:**

New occurrence of a melanoma metastasis in the gallbladder is extremely rare, especially in stable disease. The therapeutical concept must be discussed extensively in the present of this metastasized tumor.

**Conclusion:**

In otherwise stable disease, palliative surgery for metastasis in the gallbladder is a possible option to prevent biliary complications. In a palliative setting always weigh up the risks and benefits while maintaining the quality of life.

## Highlights

• Malignant melanoma metastasis into the gallbladder is a rare issue.
• Surgical treatment in a metastasized situation has to be carefully evaluated.
• Prevention of possible biliary complications is of significant importance.

## 1 Introduction

Malignant melanoma is one of the five most common types of cancer. It accounts for 4–7% of all new cancer cases and 1.9% of all cancer deaths [1]. Beside the skin, primary malignant melanoma can be found in mucous membranes, such as the lung, genitourinary or gastrointestinal tract, representing 1.3% of all melanomas [2]. In the metastasic setting, a distinction is made between locoregional metastasis in the surrounding tissue and lymph nodes (stage IIIA–D) and distant metastasis (stage IV) [3]. Traditionally, prognosis of metastatic melanoma was very poor with 10-year overall survival (OS) rate of less than 10% [4]. Single-agent chemotherapy is associated with response rates of only 5–20% [5,6]. Early approaches with the use of immunotherapeutic agents such as high-dose interleukin-2 were associated with durable responses in a small percentage of patients but with substantial toxicity [7]. Over the past 10 years, increased biological understanding and access to innovative therapeutic substances have transformed advanced melanoma into a new oncological model for treating solid cancers. Treatments that target B-Raf proto-oncogene serine/threonine-kinase (BRAF)V600 (Val600) mutations using selected BRAF inhibitors combined with mitogen-activated protein kinase inhibitors (MEK inhibitors) have significantly improved response and OS. Furthermore, advanced cutaneous melanoma has developed into a prototype for testing checkpoint-modulating agents [8]. 5-year OS-rates for metastatic melanoma have increased substantially from less than 10% to up to 40–50% today in countries that have access to these innovations [9].

Regional lymph nodes, lung and brain are the most frequent metastatic sites, involvement of the gastrointestinal system is rare with 2–4%, mostly in the small intestine, colon and stomach [10,11]. Gallbladder metastases is mainly an incidental findings in autopsies and rarely described in living patients due to poor prognosis with a mean survival rate of 8.4 months [[11], [12], [13]]. The main therapeutic goal in these patients is the prevention of complications such as cholecystitis, icterus and the obstruction of the common bile duct, haemobilia and biliary fistulas [14]. However, in a scenario of an isolated gallbladder metastasis, one year survival-rate is 100% [15]. In this case report, we intend to describe the nature of the disease, outline therapeutic options and raise awareness of the importance of follow-up in these patients. This case report has been reported in line with the SCARE criteria [16].

## 2 Case

**Fig. 1:**
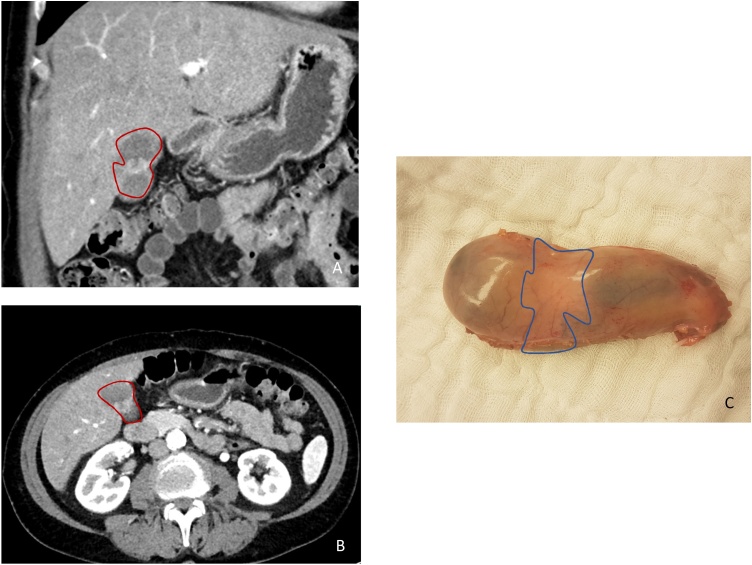
CT abdomen (portal venous phase) transversal plane (A), coronal plane (B) and macroscopic finding (C) with gallbladder (red) and metastasis (blue).

**Fig. 2:**
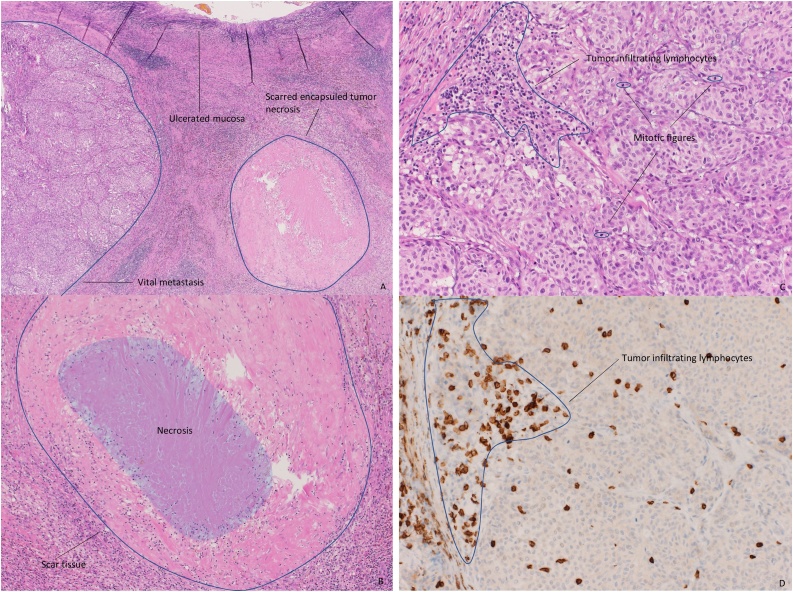
A (H&E^1^, 40×) In the overview of the gallbladder an intramural vital amelanotic metastasis and a necrotic metastasis of the melanoma surrounded by scar tissue is visible. -B (H&E, 100×) Necrotic metastasis surrounded by scar tissue and a dense lymphohistiocytic infiltrate. -C (H&E, 200×) Vital amelanotic tumor with numerous mitotic figures and tumor-infiltrating lymphocytes. -D (CD8^2^, 200×) Immunohistochemically, the tumour-infiltrating lymphocytes are cytotoxic T lymphocytes.

A 62-year-old patient with initial right sided paravertebral malignant melanoma. The slowly growing skin lesion was resected in July 2013. The findings showed a malignant melanoma Breslow 1.3 mm, stage IIA (pT2b pN0 (sn 0/2) cM0). A second resection with a safety margin of 0.5 cm was done. In February 2016, a further excision of a 6.5 mm (resection margin 0.1 mm) cutaneous lesion in the right flank was performed. Histological examination arised metastatic progression and a resection with a 0.5 cm margin was carried out. Next gene sequencing analysis of the metastasis revealed mutations in BRAFp.V600E. Further staging with Positron Emission Tomography (PET)/computed tomography (CT) revealed several cutaneous and subcutaneous masses with hypermetabolism and multiple lung metastases. Immunotherapy with the anti-PD-1 antibody pembrolizumab was initiated. After three months of therapy, CT scan in July 2016 showed a good partial remission. The patient suffered various immune-mediated toxicities, including grade 3 skin exanthema and elevation of creatinine kinase without evidene of myocarditis. The macular exanthema was biopsied and revealed an indicated Graft versus Host Disease-like lichenoid dermatitis with focal destruction of the basal cell series, dyskeratosis, discrete pigment incontinence and perivascular lymphocytosis, well compatible with checkpoint inhibitor side effects. The patient also had pronounced vitiligo. Due to severe side effects, pembrolizumab was discontinued after 3 months. Toxicities were treated with oral corticosteroids for 2 months. After this period, a re-exposure with pembrolizumab was attempted, which led to a renewed massive flare-up of skin toxicity. Therefore pembrolizumab was permanently suspended. A growing metastasis on the left dorsal lower leg was irradiated with a total of 30 Gy in December 2016. Repeated CT and Magnet Resonance Tomography scans showed size-constant pulmonary metastases with central calcifications, suggesting stable pulmonary disease for three years. In September 2018 a CT scan revealed a new lesion in the gallbladder wall adjacent to a hypervascularized liver lesion, highly suspicious of a further metastatic deposit. A biopsy of the hepatic lesion showed no sign of malignancy, however the gallbladder lesion emerged to be a metastasis of an amelanotic melanoma. Due to this disease progression, a new therapy with pembrolizumab was again initiated. This time the patient initially tolerated the treatment without relevant side effects. Radiologically treatment led to a stable disease. Following extensive discussion at our multidisciplinary team meeting (MDT), elective laparoscopic cholecystectomy was recommended on the base of otherwise stable disease over years. Intraoperatively, there was an inconspicuous situs without signs of peritoneal carcinomatosis. Macroscopically, a slight discoloration was seen on the anterior surface of the gallbladder, which revealed itself as a 1 cm intravesical tumor nodule on palpation (Fig. 1). Histopathology confirmed a largely amelanotic, partly spindle cell, partly epitheloid cell, predominantly vital metastasis. The immunohistochemical study showed PD-L1 (SP263 Ventana) positivity. Membrane staining in tumor cells and tumor-associated mononuclear immune cells corresponded to a MEL score 2 (5%) according to Daud et al. (Fig. 2A–D) [17]. Due to the renewed flare-up of the skin exanthem immediately prior to the planned surgery, another short-term therapy with prednisone was necessary. After discontinuation of the corticosteroid, the skin reaction flared up briefly. Postoperatively, it was therefore decided not to continue the therapy with pembrolizumab for the time being. The radiological follow-up showed an unchanged partial remission of the pulmonary metastases. At present, 15 months after resection of the gallbladder, the patient is in very good general condition without indications of a new tumor progression.

## 3 Discussion

Metastatic spread of a malignant melanoma into the gallbladder is a rare event, described only in individual cases. The newly emerging gallbladder lesion appears unusual since the other metastases responded well to immunotherapy. Since, the reason of the delay in the initial surgical management cannot be clearly identified, the main topic in this case, is whether surgery should be performed in this widespread metastatic situation. The decision of performing the operation was based on the fact of an otherwise stable disease over years, supported by a case report of Gogas et al. [13]. Alternatively, surgery could have been withhold until biliary complication occurred. Possible alternatives would have been antibiotics, biliary stenting or cholecystostomy.

With regards to surgical access we decided to perform a minimal invasive technique. Due to the size and clearly localized lesion on imaging, a laparoscopic procedure was possible. The open technique would be safer with respect to tumor spread in the port canal, but associated with higher morbidity [12,18].

The specimen was analyzed histopathologically and immunohistochemically. The examination of tumor cells revealed membrane-positive expression of PD-L1 in 5% of tumor cells and tumor-associated mononuclear immune cells. Melanomas with more than 1% PD-L1 positive tumor cells and tumor-associated immune cells are more likely to respond to immunotherapy than PD-L1 negative tumors [17]. Although PD-L1 expression is not regarded as a relevant predictive tumor marker, from an oncological perspective, further therapy with pembrolizumab was recommended. Alternatively, a combined BRAF and MEK inhibition, would have been a possible approach in this clinical stage. Since combination show a higher response rate and longer survival rate as compared to BRAF inhibition alone (Dabrafenib and Trametinib, Encorafenib and Binimetinib and Vemurafenib plus Cobimetinib) [[19], [20], [21]]. Of note, the patient initially had a melanotic melanoma, but the resected metastasis in the gallbladder showed an amelanotic melanoma. A dedifferentiation of the initial tumor not responding to immunotherapy is a possible explanation. In summary the therapeutic concept and management of patients with widespread metastatic melanoma needs an interdisciplinary discussion in a MDT, properly balancing risk and benefits with quality of life in these palliative situations.

## 4 Conclusion

Diagnosis of gallbladder metastasis in malignant melanoma is difficult due to its rarity and absence of symptoms. However, complications can be life limiting. Management can be surgical or medical, but discussion and definition of a major treatment plan need to be addressed in MDT meetings. The laparoscopic cholecystectomy can avoid biliary complications in a palliative setting.

## Declaration of Competing Interest

There are no conflict of interests regarding this article.

## Funding

There are no sources of funding regarding this article.

## Ethical approval

On the basis of this being a case report and with a present handwritten signed consent of the patient, this case report is exempt from ethical approval.

## Consent

Written informed consent was obtained from the patient for publication of this case report and accompanying images. A copy of the written consent is available for review by the Editor-in-Chief of this journal on request.

## Author contribution

- Dr. med. Gabriel Fridolin Hess: Concept, design, manuscript preparation, image preparation.

- Prof. Dr. med Katharina Glatz: Image preparation, histological analysis, proofreading.

- PD Dr. med. et Dr. phil. Sacha I. Rothschild: History workup, analysis of the oncological therapies, proofreading.

- Prof. Dr. med. Otto Kollmar: Proofreading.

- PD Dr. med. Savas Deniz Soysal: Proofreading.

- Prof. Daniel T. Boll: Image preparation, proofreading.

- Prof. Dr. med. Raoul André Droeser: Proofreading.

- Dr. med. Robert Mechera: Design, final proofreading.

## Registration of research studies

N/A.

## Guarantor

Dr. med. Gabriel Fridolin Hess.

## Provenance and peer review

Not commissioned, externally peer-reviewed.

## Footnotes

1 Hematoxylin and Eosin staining.

2 CD8 staining.

## References

[1] Siegel R.L., Miller K.D., Jemal A. (2019). Cancer statistics, 2019. CA Cancer J. Clin..

[2] Heusser R., Baumann A., Noseda G. (2017). Krebs in der Schweiz: wichtige Zahlen. Krebsliga Schweiz.

[3] American Cancer Society A.E., Karnell L.H., Menck H.R. (1998). Cancer. https://deepblue.lib.umich.edu/handle/2027.42/34348.

[4] Helmke B.M., Otto H.F. (2004). Das anorektale Melanom. Pathologe.

[5] Gershenwald J.E., Scolyer R.A. (2018). Melanoma staging: american joint committee on cancer (AJCC) 8th edition and beyond. Ann. Surg. Oncol..

[6] Balch C.M., Soong S., Gershenwald J.E., Thompson J.F., Reintgen D.S., Cascinelli N. (2001). Prognostic factors analysis of 17,600 melanoma patients: melanoma staging system. Society.

[7] McClay E.F., Mastrangelo M.J. (1988). Systemic chemotherapy for metastatic melanoma. Semin. Oncol..

[8] Lui P., Cashin R., Machado M., Hemels M., Corey-Lisle P.K., Einarson T.R. (2007). Treatments for metastatic melanoma: synthesis of evidence from randomized trials. Cancer Treat. Rev..

[9] Dillman R.O., Barth N.M., Vandermolen L.A., Mahdavi K., McClure S.E. (2012). Should high-dose interleukin-2 still be the preferred treatment for patients with metastatic melanoma?. Cancer Biother. Radiopharm..

[10] Schadendorf D., van Akkooi A.C.J., Berking C., Griewank K.G., Gutzmer R., Hauschild A. (2018). Melanoma. Lancet.

[11] Kandolf Sekulovic L., Peris K., Hauschild A., Stratigos A., Grob J.J., Nathan P. (2017). More than 5000 patients with metastatic melanoma in Europe per year do not have access to recommended first-line innovative treatments. Eur. J. Cancer.

[12] Meyers M., Frey D., Levine E. (1998). Pancreaticoduodenectomy for melanoma metastatic to the duodenum: a case report and review of the literature. Am. Surg..

[13] Gogas J., Mantas D., Gogas H., Kouskos E., Markopoulos C., Vgenopoulou S. (2003). Metastatic melanoma in the gallbladder: report of a case. Surg. Today.

[14] Haskaraca M.F., Ozsoy M., Ozsan I., Kurt K. (2012). Primary malignant melanoma of the gallbladder: a case report and review of the literature. Case Rep. Surg..

[15] Marone U., Caracò C., Losito S., Daponte A., Chiofalo M.G., Mori S. (2007). Laparoscopic cholecystectomy for melanoma metastatic to the gallbladder: is it an adequate surgical procedure? Report of a case and review of the literature. World J. Surg. Oncol..

[16] Agha R.A., Borrelli M.R., Farwana R., Koshy K., Fowler A.J., Orgill D.P. (2018). The SCARE 2018 statement: updating consensus Surgical CAse REport (SCARE) guidelines. Int. J. Surg..

[17] Daud A.I., Wolchok J.D., Robert C., Hwu W.-J., Weber J.S., Ribas A. (2016). Programmed death-ligand 1 expression and response to the anti-programmed death 1 antibody pembrolizumab in melanoma. J. Clin. Oncol..

[18] Katz S., Bowne W., Wolchok J., Busam K., Jaques D., Coit D. (2007). Surgical management of melanoma of the gallbladder: a report of 13 cases and review of the literature. Am. J. Surg..

[19] Ribas A., Daud A., Pavlick A.C., Gonzalez R., Lewis K.D., Hamid O. (2019). Extended 5-Year Follow-up Results of a Phase Ib Study (BRIM7) of Vemurafenib and Cobimetinib in BRAF-Mutant Melanoma.

[20] Dummer R., Ascierto P.A., Gogas H.J., Arance A., Mandala M., Liszkay G. (2018). Encorafenib plus binimetinib versus vemurafenib or encorafenib in patients with BRAF-mutant melanoma (COLUMBUS): a multicentre, open-label, randomised phase 3 trial. Lancet Oncol..

[21] Larkin J., Ascierto P.A., Dréno B., Atkinson V., Liszkay G., Maio M. (2014). Combined vemurafenib and cobimetinib in BRAF-mutated melanoma. N. Engl. J. Med..

